# Protein-Based Fiber Materials in Medicine: A Review

**DOI:** 10.3390/nano8070457

**Published:** 2018-06-22

**Authors:** Kelsey G. DeFrates, Robert Moore, Julia Borgesi, Guowei Lin, Thomas Mulderig, Vince Beachley, Xiao Hu

**Affiliations:** 1 Department of Physics and Astronomy, Rowan University, Glassboro, NJ 08028, USA; defratesk6@students.rowan.edu (K.G.D.); moorer1@students.rowan.edu (R.M.); ling8@students.rowan.edu (G.L.); 2 Department of Biomedical Engineering, Rowan University, Glassboro, NJ 08028, USA; borgesij5@students.rowan.edu (J.B.); beachley@rowan.edu (V.B.); 3 Department of Mechanical Engineering, Rowan University, Glassboro, NJ 08028, USA; mulderigt9@students.rowan.edu; 4 Department of Molecular and Cellular Biosciences, Rowan University, Glassboro, NJ 08028, USA

**Keywords:** protein, nanofibers, biomaterials fabrication, medicine, tissue engineering, wound healing, drug delivery

## Abstract

Fibrous materials have garnered much interest in the field of biomedical engineering due to their high surface-area-to-volume ratio, porosity, and tunability. Specifically, in the field of tissue engineering, fiber meshes have been used to create biomimetic nanostructures that allow for cell attachment, migration, and proliferation, to promote tissue regeneration and wound healing, as well as controllable drug delivery. In addition to the properties of conventional, synthetic polymer fibers, fibers made from natural polymers, such as proteins, can exhibit enhanced biocompatibility, bioactivity, and biodegradability. Of these proteins, keratin, collagen, silk, elastin, zein, and soy are some the most common used in fiber fabrication. The specific capabilities of these materials have been shown to vary based on their physical properties, as well as their fabrication method. To date, such fabrication methods include electrospinning, wet/dry jet spinning, dry spinning, centrifugal spinning, solution blowing, self-assembly, phase separation, and drawing. This review serves to provide a basic knowledge of these commonly utilized proteins and methods, as well as the fabricated fibers’ applications in biomedical research.

## 1. Introduction

Fibrous materials, so often used in industrial applications and the textile industry, have now migrated into biomedical research. To date, polymer-based fibers with diameters on the micro- or nanoscale have been explored in drug delivery [1,2,3,4], wound healing [5,6,7], tissue engineering [8,9,10], and biosensor technologies [11,12,13] due to their high surface-area-to-volume ratio, mechanical strength, porosity, potential for surface modification, and tunability [13,14,15]. Equally important in biomaterials engineering, however, is the need for materials to be both biocompatible and biodegradable. Therefore, to maximize these properties, natural polymer-based fibers made from proteins have begun to be developed [16,17,18,19].

The appeal of protein-based fibers for biomedical applications stems from the fact that many proteolytic enzymes capable of degrading commonly used natural polymers are already present in the body. In the case of protein-based biomaterials, degradation of these materials leads to the production of amino acids that pose no risk of toxicity and can be reabsorbed by the body [20,21]. In the field of tissue engineering, this avoidance of toxic byproducts is of particular importance since materials must degrade and be replaced by native tissue to achieve complete regeneration [22]. In the field of drug delivery and nanomedicine, protein-based nanofibers may have the ability to store pharmaceutical products and biological molecules without threatening their bioactivity [23,24,25]. Their controllable degradation through crosslinking or post-fabrication modifications has also been shown to allow for the controllable release of drugs, with no added toxicity from material byproducts during fabrication [26,27]. Blending of proteins with other natural and synthetic polymers can also allow for the development of versatile materials with modifiable degradation and physical properties [28,29]. The biodegradable nature of protein-based fiber materials also supports the efforts of green and sustainable engineering. Such applications reduce the dependence on petroleum-based polymers avoiding the pollution issues caused by the disposal of these materials and their byproducts [30]. Additionally, many proteins, such as silk, soy, and corn zein, are very abundant and easy to isolate [31].

The incorporation of natural polymers in biomaterials has also been shown to enhance cell attachment due to the presence of native cell attachment motifs [25,32]. Thus, the use of protein-based fibers in tissue engineering and nanomedicine has both medical and commercial appeal. Despite these advantages, however, standardization of the mechanical and physical properties of protein-based fibers remains challenging. Such materials have been shown to vary depending on the method of fiber production, the fiber diameter, and the composition of the fiber [33,34,35]. In order to illustrate the appeal of protein-based fibers and regulate their use, this review serves to provide a basic knowledge of the commonly used materials and methods for the fabrication of protein-based fibers and their corresponding use in tissue engineering, wound healing, and drug delivery. Popular proteins, such as keratin, collagen, silk, elastin, zein, and soy, are given particular attention, as well as current fabrication methods, including electrospinning, wet/dry jet spinning, dry spinning, centrifugal spinning, solution blowing, self-assembly, phase separation, and drawing.

## 2. Protein Materials

**Figure 1:**
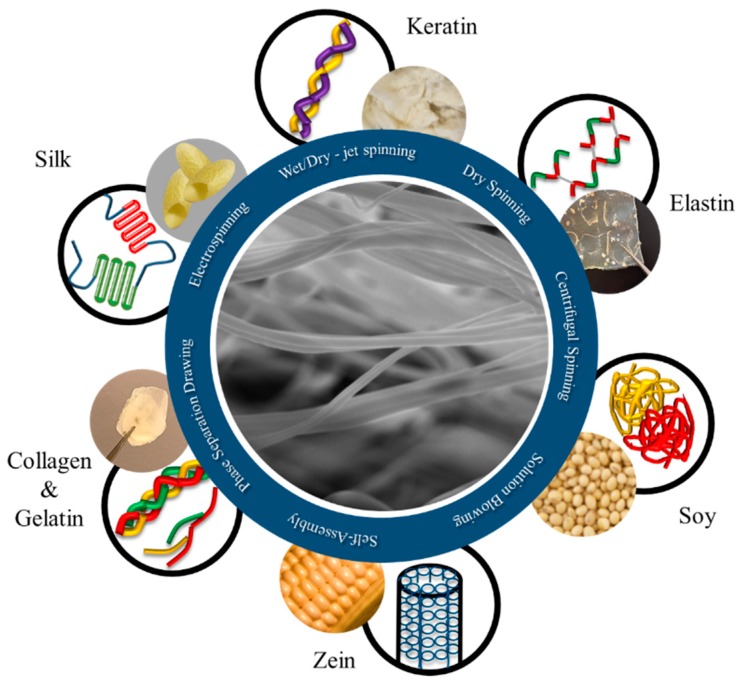
Protein-based biomaterials can be made from a variety of sources. The origin and structures of some of the most commonly used proteins are shown. These include collagen or gelatin, silk, keratin, elastin, soy, and corn zein. These proteins can then be processed into fibers with unique physical properties through a variety of methods.

Some of the specific types of proteins that will be discussed include keratin, collagen, silk, elastin, zein, and soybean (Figure 1). These proteins are some of the most common protein polymers used for the fabrication of fibers for biomedical application.

### 2.1. Elastin

Elastin is a naturally-occurring protein found in the extracellular matrix (ECM) that maintains the elasticity of connective tissue in the human body. Tropoelastin, the 72 kDa precursor to elastin, is first synthesized by cells in the rough endoplasmic reticulum and consists of alternating hydrophobic and hydrophilic domains. The hydrophilic domains contain lysine residues interspersed by alanine residues, and this arrangement allows for tetrafunctional crosslinking of tropoelastin molecules by the lysine oxidase enzyme. Crosslinking of tropoelastin molecules is further strengthened by self-assembly of the hydrophobic domains that consist of repeating motifs of non-polar residues of glycine, valine, and proline [36]. Complete assembly of elastin molecules occurs outside of cells due to the protein’s large size. Tropoelastin molecules are believed to align and crosslink after interacting with extracellular microfibrils near the cell surface. While these microfibrils provide an integral framework for elastin assembly, as elastin-rich tissue forms, these microfibrils become detectable only on the periphery of the protein structures [37]. The elastin-based fibers are then arranged in a variety of structures depending on the tissue’s location. For example, in ligaments, elastin fibers are arranged in parallel-oriented structures, but can be found in a honeycomb-like pattern in cartilage [36].

In biomaterials engineering, elastin is used to describe a variety of elastic proteins and peptides rather than one single molecule. It can be used in a variety of forms that include, most commonly, soluble elastin [18,38,39], recombinant tropoelastin [40,41], and synthetic elastin-like peptides that may also be hybrids with other proteins, such as silk [42,43,44,45]. The inclusion of elastin in nanofiber scaffolds has been shown to increase fiber elasticity and provide for better cell attachment [18]. Due to elastin’s elasticity and resilience, it has found special purpose in the development of vascular grafts, since fibrous scaffolds made from the protein have been shown to closely match the compliance of natural arteries [46,47,48].

### 2.2. Collagen and Gelatin

Collagen is a fibrous protein that serves as the main component of the ECM. While the majority of collagen found in body can be classified as type I, II, or III, as many as 29 different types of collagen have been identified. All collagen exhibits a repeating X-Y-Gly amino acid sequence, where glycine is always present as the third residue. X and Y can denote any amino acids, with proline and hydroxyproline being the most common ones. The glycine residue allows for a stable secondary structure formation of collagen, which consists of three strands coiled around each other to form a triple helix. These triple helices can then arrange into different quaternary structures depending on the type of collagen. In fibrillar types, such as types I–III, V, and XI, the coiled coils are crosslinked by the lysine oxidase enzyme to form fibrils that then aggregate to form fibers [49]. The spaces between collagen crosslinking domains measures 67 nm and these gaps give collagen its striated appearance [50].

Due to its natural abundance, Type I collagen is the most common class of collagen used in biomaterials development [49]. The production of collagen fibers has allowed for the generation of biomimetic tissue engineering scaffolds that closely resemble the natural ECM. Therefore, fiber meshes have been used for bone [51,52,53], cartilage [54], vasculature [55,56], ligament [57,58], skin [59,60], muscle [61], and nerve [62,63] regeneration. These materials allow for cell attachment, penetration, and proliferation due to collagen’s ability to interact with cell surface receptors, such as the α2β1, α1β1, α10β1, and α11β1 integrins [64]. The mechanical properties of collagen-based fiber meshes can also be easily modified by chemical or physical crosslinking, although this has been shown to affect their biocompatibility [65].

Due to its complicated hierarchical structure, collagen fibrils can be difficult to extract and isolate. However, the coiled coil can be easily broken down through hydrolysis to produce three polypeptide strands, known as gelatin [66]. These strands can be further degraded into shorter amino acid sequences by matrix metalloproteinases, making gelatin a biodegradable material with low immunogenicity. Due to the presence of alkaline and acidic amino acid residues, gelatin is also amphoteric and can form a thermally reversible network in water [67]. Like collagen, the mechanical properties of gelatin-based materials can be further modified through chemical or physical crosslinking, which is often necessary due to the instability of the natural biopolymer in water at body temperature [68]. Crosslinked gelatin-based materials and fibers made by dissolving gelatin in polar solvents to prevent aggregation [69] have been shown to promote ocular [70,71], bone [72,73], cardiovascular [74,75], nerve [76,77], and skin [78] regeneration.

### 2.3. Silk

Silk is a natural biopolymer produced by insects, spiders, and worms that consists of two main proteins. Silk sericin, the sticky protein found on the outside of silk strands, makes up 15–35% of silk cocoons and must be removed through a degumming process to extract the more versatile silk fibroin protein [79]. The particular amino acid sequence of silk fibroin can vary depending on its species, but is predominately composed of hydrophobic blocks composed of glycine, alanine, and serine residues and hydrophilic blocks consisting of charged amino acids. The hydrophobic blocks allow for the formation of β-sheets within the protein, giving silk high tensile strength, while the hydrophilic blocks give silk fibroin its elasticity [80]. Variations in specific sequences account for differences in the secondary structure of silk, which, in turn, affects its mechanical properties, thermal stability, chemical characteristics, and solubility [79,81].

Silk fibroin obtained from the *Bombyx mori* silkworm is one of the most commonly used biomaterials due to its availability and low cost [29,79]. It has been shown to exhibit excellent biocompatibility, bioactivity, biodegradability, tunability, mechanical stability, and low immunogenicity, allowing silk-based fibers to be used to create tissue engineering scaffolds that allow for bone [82,83,84,85], cartilage [86], heart valve [87], and nerve [88] regeneration. The oxygen and water vapor permeability of silk also encourages its use in wound healing [25,89]. The mechanical properties and stability of silk-based biomaterials can also be modified through methanol treatments that increase β-sheet crystallinity and strength [29,90].

In addition to silk produced from worms, dragline silk produced by the *Nephila clavipes* spider has found use in biomaterials development. Like silkworm silk, it has shown low immunogenicity, high tensile strength, and biodegradibility. Recent studies have outlined the dragline silk’s ability to promote cell adhesion, migration, and proliferation of dental pulp stem cells [91] and cardiomyocytes [92], showing its promise as a component in tissue engineering scaffolds.

### 2.4. Keratin

Keratin is an insoluble structural protein that makes up the bulk of the adnexa of the epidermis, including hair, horns, and fingernails. The protein can be further characterized a soft or hard keratin depending on its amino acid sequence. Both soft and hard keratin, however, have similar secondary structures that consist of two chains, each containing a central alpha-helical domain. These chains are designated as type I and II and interact to form heterodimers that polymerize to form filaments [93]. Some forms of keratin, like that found in hair, have a high content of cysteine residues that interact through disulfide bonding, enhancing the mechanical strength of the protein [94]. Extraction of keratin requires disruption of these disulfide bonds. This can be accomplished through an oxidation of the protein [95].

The presence of cell binding motifs on keratin, as well as its ability to self-assemble, make it an ideal natural polymer to be used in the creation of biomaterials for tissue regeneration [93]. However, because keratin is known to exhibit poor mechanical stability, it is often combined with other natural or synthetic polymers to create composite fibers [96,97]. Such composites have been used for the skin [98], cartilage [99], and bone [97,100] tissue regeneration.

### 2.5. Zein

Zein is the major storage protein in corn and is a member of the prolamin group of proteins. Its structure and solubility are dictated by its amino acid sequence, which primarily consists of non-polar, uncharged residues, such as glutamine, leucine, proline, and alanine. In addition to its biodegradability and biocompatibility, recent studies suggest that corn zein can exhibit anti-oxidative and antimicrobial properties [101,102]. These properties have led to zein’s expanded use in biomedical engineering.

Studies have shown that the corn protein is compatible with human umbilical vein endothelial cells, human hepatocytes, and mice fibroblasts [103]. Neat zein nanofibers have been shown to exhibit low mechanical strength and stability, and the high hydrophobicity of the protein may also prevent cell attachment [104,105,106]. Therefore, it is often necessary to incorporate additional synthetic or natural polymers and chemical crosslinking to create successful tissue engineering scaffolds. Studies suggest that these composites may promote successful tissue regeneration when used as a scaffold [107,108]. While it may be counter to cell attachment, the hydrophobicity of corn zein does enhance its capabilities as a drug delivery vehicle since it is more resistant to hydrolysis, allowing for longer, more sustained release of pharmaceuticals [109,110].

### 2.6. Soybean Protein

Soybean protein is a globular protein composed of two main subunits referred to as conglycinin 7S and glycinin 11S. Both subunits contain regions of non-polar amino acids, such as alanine, valine, and leucine; basic amino acids, including lysine and arginine; and non-charged polar residues, like cysteine and glycine. The globular structure of soybean protein makes it resistant to hydrolysis and incredibly stable, leading to its long shelf-life [111]. For biomaterials engineering, the protein is of particular interest due its abundance of functional groups that allow for surface modification and blending with other polymers [112].

Soy protein is biodegradable and can be obtained from abundant renewable resource. In recent years, soybean products, such as soybean whole fat (SF), soy protein concentrate (SPC), and soy protein isolate (SPI), have become alternatives to petroleum polymers due to their abundance and adhesive properties [113]. Compared to other plant protein-based membranes, SPI-based materials are clearer, smoother, more flexible, and have impressive gas barrier properties compared to lipid and polysaccharide formulations.

Although the solubility of soybean protein is relatively low in acidic solutions, solvents with higher pH above 4.8 have been used to process soybean, allowing for fiber fabrication [114]. Due to the presence of ECM-mimetic peptides within the protein, such fibers have seen great success as tissue engineering scaffolds [111]. Some of the most common applications of soy protein materials include skin regeneration and wound healing [115,116,117].

## 3. Fabrication Methods

There are numerous ways of fabricating protein-based fiber materials. Table 1 lists fabrication methods along with controlled parameters that affect fiber properties.

### 3.1. Electrospinning

**Figure 2:**
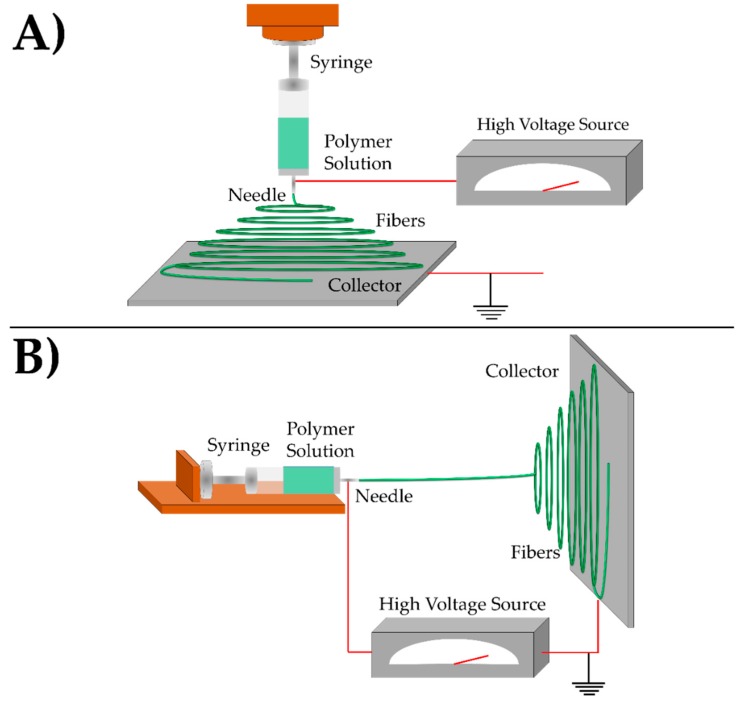
Schematic showing the set-up of a (**A**) vertical electrospinning system and (**B**) horizontal electrospinning system.

Electrospinning is a nanofiber fabrication method that consists of three main components: a polymer solution within a metal tipped syringe, an applied high voltage, and a grounded collector [131,132,133,134,135,136,137]. Figure 2 shows two common electrospinning systems (vertical and horizontal electrospinning systems) utilized in current research. In the vertical system, gravity is an important parameter for controlling fiber formation, while horizontal spinning system relies mainly on the electrical force between the spinning device and the collector.

**Figure 3:**
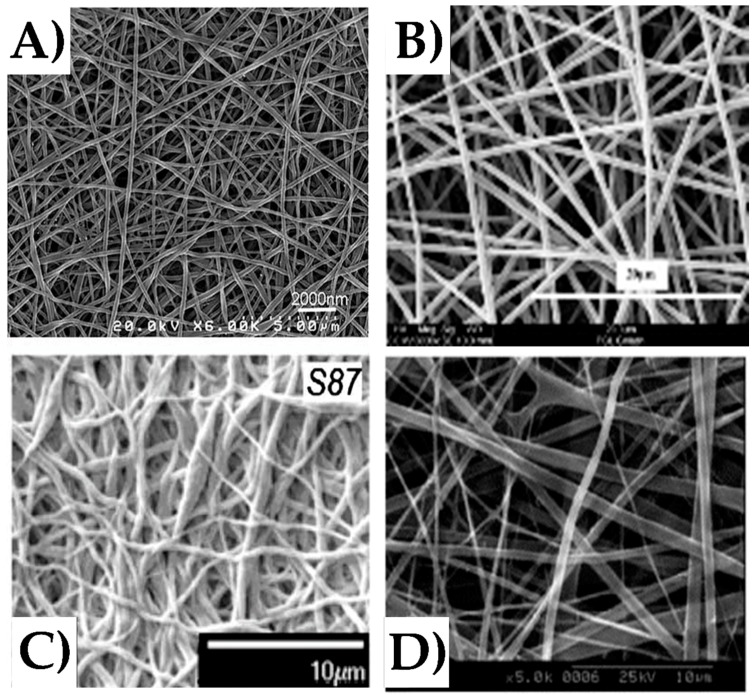
Scanning electron microscope images of (**A**) pure silk nanofibers, (**B**) PCL-gelatin nanofibers, (**C**) silk-PEO nanofibers, and (**D**) type I collagen nanofibers fabricated using the electrospinning technique (reproduced with permission from [19,78,136,139], copyright Elsevier, 2017 (**A**); Elsevier, 2007 (**B**); John Wiley and Sons, Inc., 2010 (**C**); Elsevier, 2006 (**D**)).

Before the electrospinning process, polymers are dissolved into a solvent and the solution is placed inside the syringe. To begin the process, the solution is forced out of the syringe at a constant flow rate. Simultaneously, a high voltage is applied to the solution, resulting in repulsive interactions between like charges within the solution. A Taylor cone [138] is formed at the end of the syringe when the electrical forces and surface tension forces in the solution are at equilibrium. At a critical value, the electrical forces overcome the surface tension forces and a jet of solution propels out of the Taylor cone and towards the grounded collector. At ideal conditions, as the solution jet travels, the solvent evaporates from the solution, leaving non-woven, polymer fibers due to high surface area to volume ratio, and finally gathered on the collector [131,132,133,134,135,136,137]. Fibers are produced with diameters in the range of 10 nm–10 µm, and various collector modifications can also allow the formation of aligned nanofiber arrays and non-woven yarns. In the literature, numerous protein nanofibers have been fabricated using the electrospinning technique. Generated fiber mats for silk, collagen, and gelatin-based fibers are shown in Figure 3 at various scales.

Electrospinning is a simple, reliable process that produces fibers with controllable properties. The process is able to produce a versatile range of fibers, including polymer-, synthetic-, and composite-based fibers [131,134]. The properties of the fibers can be influenced by controlling different parameters. These parameters can be categorized into three types: solution, process, and ambient [131,134]. Table 1 lists controllable parameters in their respective categories. Reproducibility and functionalization of protein-based nanofibers may be enhanced by treating the protein prior to fabrication. This approach was adopted by Pegg et al. [140] to produce alginic acid nanofibers. Prior to spinning, the alginic acid was converted to ammonium alginate by reacting the polymer with amine-containing cargo. This pre-treatment allowed for more uniform functionalization and enabled the fibers to carry diverse therapeutics, such as lidocaine, neomycin, and papain. Electrospun nanofibers like these are also useful in a variety of applications due to their high surface-area-to-volume ratio [131,134] and modifiable surface porosity [134].

### 3.2. Wet/Dry-Jet Spinning

**Figure 4:**
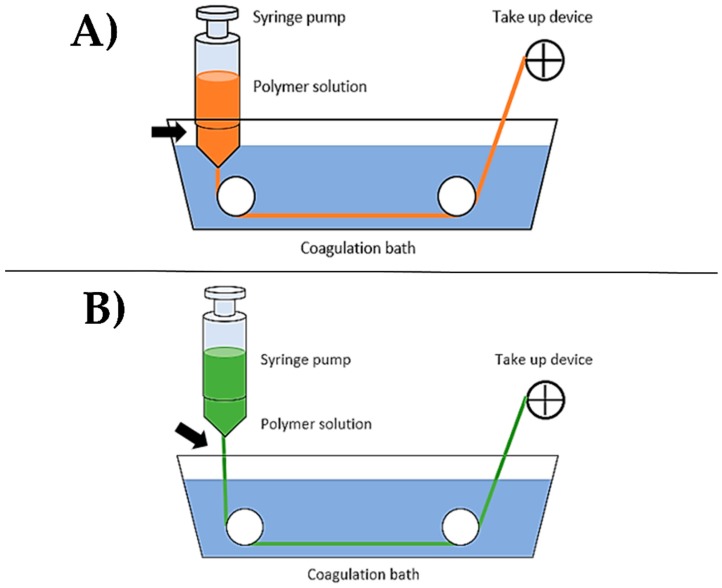
Common systems of (**A**) wet spinning, and (**B**) dry-jet wet spinning. In dry-jet wet spinning, the polymer solution is extruded through an air gap before the coagulation bath, resulting in higher molecular alignment compared to conventional wet spinning [124,141,142].

Wet spinning is a fiber fabrication method that consists of a polymer solution, a spinneret, and a coagulation bath (Figure 4A). During the fabrication process, the polymer solution is extruded via a syringe pump through the spinneret directly into a coagulation bath. Polymer fibers form in the coagulation bath as the solvent is removed either through chemical reaction or diffusion [141]. After formation, the remaining fiber material is collected and dried. Drawing, or applying tension to the fibers can occur immediately after the spinneret [141], during drying [125], or further down the spinning line [142,143] to elongate the fiber, increase molecular alignment and, consequently, stiffness and strength. Wet spinning fabrication systems may implement multiple drawings or baths in order to improve molecular alignment and orientation.

A modified version of wet spinning, referred to as dry-jet wet spinning has been developed (Figure 4B) [124,141,142]. In dry-jet wet spinning, the polymer solution is extruded through an air gap before the coagulation bath, rather than directly into the bath. Studies have shown that dry-jet wet spinning can result in greater molecular alignment compared to conventional wet spinning [142].

By controlling parameters, such as the diameter of the spinneret, polymer solution concentration, and flow rate, fiber properties, such as diameter, orientation, and morphology [122,125,141,143], can be modified. Unlike in electrospinning, the fibers are not exposed to a high voltage that may denature natural polymers, such as proteins. Additionally, drawing of the fibers after formation can lead to enhanced material properties due to higher molecular alignment. However, the wet/dry spinning methods typically produces only micron-sized fibers, while electrospinning is a common method of producing nanofibers.

### 3.3. Dry Spinning

**Figure 5:**
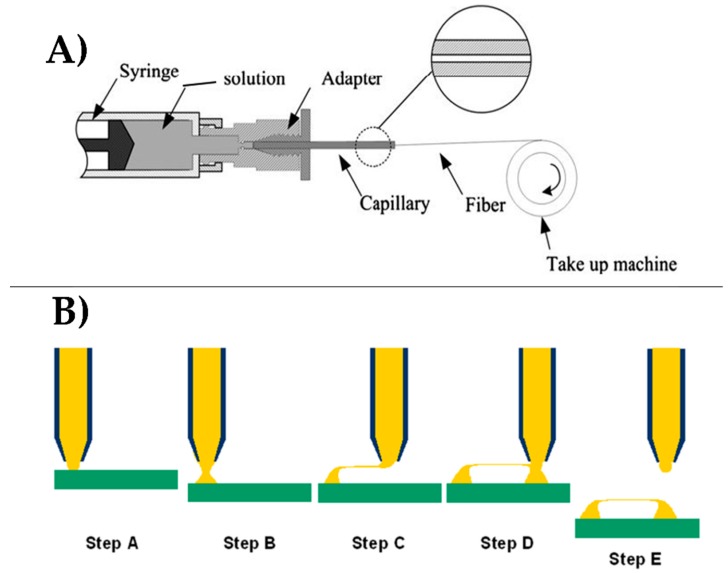
(**A**) Scheme of a typical dry spinning system. (**B**) Representation of drawing mechanism for polymer-based fiber fabrication (reproduced with permission from [126,146], copyright Elsevier, 2011 (**A**); AIP Publishing, 2006 (**B**)).

Dry spinning is a fiber fabrication method that consists of a polymer solution, a syringe, and a collector. Figure 5A shows the scheme of a typical dry spinning system. Unlike other solution spinning methods, such as electrospinning and wet spinning, dry spinning involves a single extrusion step. A key part of the method is that the polymer/protein solution is created such that the solvent of the solution will evaporate in the ambient environment during spinning. During the process, the solution is pumped through a syringe and spinneret. Ideally, the solvent of the solution will evaporate out of the solution, leaving only the polymer fiber to be collected. The fibers are then collected via a take-up device similar to those used in the wet spinning processes [126]. Similar to wet spinning, additional drawing [144], heating, or drying [126] can occur to increase the mechanical properties or ensure fiber stability.

The mechanical properties of the fiber can be affected controlling take up speed, length to diameter ratio of the spinneret, environment or temperature, and spinning rate [126,145]. Additionally, subjecting the dry-spun fiber to a post-treatment agent or post-formation drawing can enhance mechanical properties [126].

### 3.4. Centrifugal Spinning

Centrifugal spinning is a process commonly used in the industrial production of fiberglass. More recently the process has gained traction as a fabrication method of polymer fibers [129]. It exhibits some significant advantages over the more commonly practiced electrospinning method. Namely, its comparatively high production rate and its lack of dependence on a voltage resulting in greater safety [128,129]. A biopolymer solution or melt can be placed in a rotating head with a small opening referred to as the nozzle. When the head is rotated at a speed that exerts a centrifugal force on the solution or melt higher than its surface tension, the solution or melt will emerge from the nozzle as a liquid jet. The liquid jet is stretched by the combination of the centrifugal force and the air friction force and deposited into a collection area. Solidified fibers with diameters ranging from hundreds of nanometers to tens of micrometers are produced upon the evaporation of the solvent [129,147]. A process of nozzle-free centrifugal spinning has been tested by Weitz et al., resulting in fibers as small as 25 nm in diameter [148]. The viscosity, surface tension, molecular structure, molecular weight, polymer concentration, solvent structure, solvent evaporation rate, and additive of the polymer solution or melt all contribute to the morphology of the fibers, with viscosity and surface tension being the largest influences [128,129]. Rotational speed, head diameter, nozzle diameter, and distance between the nozzle and collector also largely influence the morphology of the fibers produced [129].

### 3.5. Solution Blowing

Solution blowing is a relatively new method of fiber fabrication. It involves an apparatus consisting of two concentric nozzles. A biopolymer solution is pumped through the inner nozzle as a high velocity gas flows through the outer nozzle. The flow of gas stretches the solution and ejects it from the apparatus. Fibers are formed in the air as the solvent evaporates before reaching a collector [149]. The high velocity gas is supplied from a source of compressed gas such as nitrogen, argon, or air equipped with a pressure regulator and connected to the apparatus. Biopolymer type, concentration, injection rate, gas flow pressure, and working distance all influence the properties of the fibers being produced [130].

### 3.6. Self-Assembly

Molecular self-assembly is a process ubiquitous in natural biological systems. Structures formed by self-assembly are governed by non-covalent forces, such as hydrogen bonding, electrostatic interactions, van der Waals interactions, hydrophobic interactions, stacking interactions, and water-mediated hydrogen bonding [150,151]. These non-covalent bonds between small molecules result in supramolecular architectures, such as nanofibers. The shapes and properties of resulting fibers are determined by the molecules and non-covalent bonds structuring them [152]. The driving intermolecular interactions can be influenced by environmental factors, such as salt concentration, pH, temperature, and surface characteristics [153].

### 3.7. Phase Separation

Phase separation is a rather simple process. However, it is limited to the scale of a laboratory setting [152]. The process begins with the dissolution of a protein polymer in a solvent. The temperature of the solution is then reduced to the gelation temperature, which is the point at which the solution forms a gel. Solvent exchange is carried out by immersing the gel in distilled water. When removed, the gel is blotted with filter paper and freeze-dried, resulting in the formation of a nanofibrous matrix [152]. By adjusting factors, such as gelation temperature and biopolymer concentration, the morphology of the fibers is able to be controlled [154].

### 3.8. Drawing

The commonly used fabrication method, electrospinning, produces a layer of fibers on a flat collector [155], while the fabrication method known as drawing only produces one fiber at a time. The production of single fibers limits the use of this method to the laboratory scale [152]. In the drawing process, a sharp-tipped probe is placed in contact with a droplet of biopolymer solution and withdrawn at a predetermined speed. The solvent evaporates due to the high surface area in relation to the volume. The end of the resulting fiber attached to the probe can then be connected to another droplet to form a suspended fiber [146]. The drawing process relies on the viscoelasticity of the solution so that it can maintain cohesion under the stresses of being pulled [156]. Multiple fibers can be drawn from each droplet [152]. When too much time is allowed to pass between the deposition of the solution droplet and the drawing of the fiber, the droplet will become too viscous due to the evaporation of the solvent. Furthermore, the continual shrinkage of the droplet affects the diameter of the fibers produced and limits the continuous drawing of fibers. A modification to this method, implementing the use of micropipettes, can improve the continuous formation of short fibers and provide greater control of the parameters that affect fiber properties. In this method the solution is continuously pumped through the micropipette. The droplet is formed at the tip of the micropipette and brought in contact with a substrate that it will adhere to. It is then laterally drawn before coming in contact with the substrate once again, forming a suspended fiber [146].

## 4. Applications of Protein-Based Nanofibers in Tissue Regeneration and Nanomedicine

Many protein-based fiber materials have applications in the biomedical field. These materials have been put to use, in part, because of their favorable biocompatible and biodegradable characteristics. For instance, natural protein fibers, such as keratin, silks, or collagens, are all of interest to researchers due to their mechanical properties and ability to maintain a low host immune response [157]. Protein-based fiber materials are often used for scaffolds, sutures, wound healing, ligament replacement, and drug delivery technology. Examples of these applications are summarized in Table 2 at the end of this section.

### 4.1. Tissue Engineering and Regenerative Medicine

Tissue engineering has become a major focus in the biomedical engineering community due to the lack of tissue transplants and host rejection of foreign tissue [158]. To be successful, two components must be optimized in tissue engineering—the cells and the scaffold. The scaffold is necessary as it provides the specific architecture and mechanical structure of the desired tissue by closely recapitulating the natural ECM [158,159].

**Figure 6:**
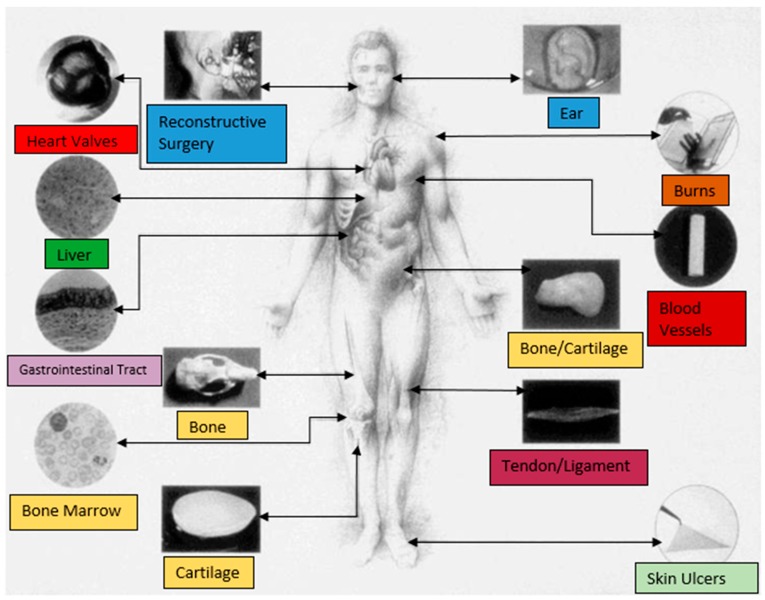
Generalized model of potential tissue engineering and medicine applications for various organ systems. (Reproduced with permission from [158], Copyright John Wiley and Sons, Inc., 1998).

Protein-based nanofiber membranes can provide an excellent scaffold in tissue engineering due to the biocompatibility, biodegradability, and tunability of the fibers. The presence of innate cell adhesion sites and functionality of these constructs also suggests the superiority of protein-based materials over those made from synthetic polymers when creating scaffolds for tissue engineering. These scaffolds create a platform for seeding cells in a defined structure, such that it mimics the host morphology, to catalyze the growth of new specialized tissues. Fibrous membranes also allow for the development of porous scaffolds, essential for cell migration, gas exchange, diffusion of nutrients, cell communication, and the elimination of waste, enhancing of the growth of the native ECM and the proliferation of surrounding cells [159]. Figure 6 maps out the different areas of the human body that have a need for tissue engineering or regenerative medicine applications.

Due to its abundance in the ECM, collagen fibers are most commonly used for the creation of tissue engineering scaffolds. Studies have successfully used these constructs for 3D cell culturing, vascular regeneration, skin grafts, bone tissue engineering, cartilage repair, nerve regeneration, spinal cord healing, and corneal defect correction [160,161,162,163,164,165,166]. For example, using the electrospinning method, Ribeiro et al. [167] developed collagen nanofiber meshes with an average fiber diameter of 30 nm. During spinning, nanohydroxyapatite crystals were also deposited onto the fibers by simultaneous electrospraying. Nanofibers were crosslinked with *N*-ethyl-*N*′-(3-dimethylaminopropyl) carbodiimide/*N*-hydroxy succinimide, allowing for the creation of a scaffold that closely recapitulated the native ECM of bone tissue and allowed for osteoblast adhesion and proliferation.

**Figure 7:**
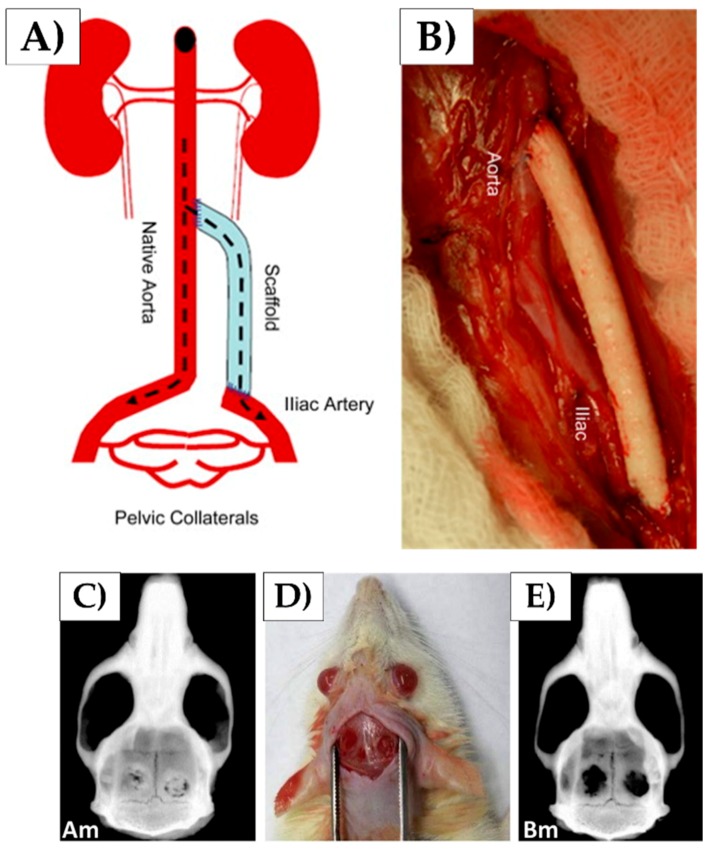
(**A**,**B**) Aortoiliac bypass using a PCL-collagen scaffold; (**C**–**E**) Effects of mulberry *B. mori* silk and non-mulberry *A. mylitta* silk on bone regeneration (reproduced with permission from [168,170], Copyright Elsevier, 2009 (**A**,**B**); Elsevier 2016 (**C**–**E**)).

Collagen-based nanofibers have also been co-fabricated with synthetic polymers to enhance the mechanical properties of the scaffolds. For example, as seen in Figure 7A,B, Tillman et al. created an electrospun PCL-collagen scaffold for a rabbit aortoiliac bypass [168]. The scaffold supported cell growth and was able to withstand normal physiological conditions. Additionally, it supported adhesion and growth of vascular cells, which was important for nutrient delivery and functionality of the implanted scaffold. Lastly, it maintained its structural integrity for over one month during the experiment. Once it was removed, the scaffold displayed biomechanical strength, comparable to its intended native artery. Ekaputura et al. also used similar collagen-PCL composite nanofibers encased in a hyaluronic acid hydrogel to promote vascularized bone regeneration through the release of vascular endothelial growth factor and platelet-derived growth factor [169].

Often, synthetic polymers are incorporated into protein-based fibrous materials to improve mechanical stability. However, proteins, such as elastin, have been incorporated into synthetic polymer-based scaffolds to modulate their mechanical properties. Foraida et al. [18] covalently conjugated elastin onto the surface of electrospun poly-lactic-co-glycolic acid (PLGA) nanofiber scaffolds through 1-ethyl-3-(3-dimethylaminopropyl) carbodiimide/*N*-hydroxysulfosuccinimide (EDC/NHS) chemistry. While this was found to improve the wettability of the scaffolds, it had little effect on their elasticity. Therefore, elastin and PLGA were also blended prior to electrospinning to create elastin-PLGA composite meshes containing fibers with an average diameter of approximately 300 nm. The incorporation of elastin was found to greatly increase the compliance of the fiber meshes. Compared to PLGA fibers with a Young’s Modulus of 4.29 MPa, the modulus of elastin-PLGA fibers was 0.59 MPa. As a result, elastin-PLGA nanofibers were able to support apical polarization and self-organization of epithelial cells, allowing for controllable cell proliferation and a higher degree of cell-cell contact compared to PLGA fibers. These characteristics better recapitulate the native arrangement of epithelial cells. As a result, elastin was identified as an integral part of tissue engineering scaffolds that hope to promote regeneration of epithelial constructs, like salivary glands.

In addition to collagen and elastin, fibrous proteins, such as silk, have been used to create tissue engineering scaffolds. Figure 7C–E depicts an in vivo rat study reported on by Melke et al. to assess the capacity of scaffolds made from mulberry *B. mori* silk and non-mulberry *A. mylitta* to induce bone regeneration in a cranial defect model. Since mylitta silk contains the natural RGD (Arg-Gly-Asp) motif, it allowed for enhanced cell adhesion and proliferation, leading to the regeneration of a higher bone volume [170]. Kim et al. has also used silk nanofiber meshes to induce bone regeneration. These meshes were also seeded with stem cells and evaluated after 31 days. They found their stem cell-seeded scaffolds and meshes were able to guide differentiation and promote new bone formation [171].

Due to its mechanical strength, silk is an ideal protein for tissue regeneration. Recently, Du et al. [87] also used silk fibroin to produce nanofibrous scaffolds for heart valve tissue engineering, but combined the natural polymer with poly(ester-urethane) (PEUU) to improve the fracture resistance of the scaffold. These scaffolds were created by combining the two polymers in hexafluoroisopropanol and following the electrospinning technique previously highlighted. Meshes consisting of randomly-orientated fibers with varying diameters were created. Average diameter and the hydrophilicity of the scaffold were dependent upon the ratio of silk fibroin to PEUU, both of which decreased with the addition of PEUU. However, these changes did not significantly affect cell adhesion and proliferation, illustrating that the mechanical properties of silk materials can be easily optimized through the addition of synthetic polymers without sacrificing biocompatibility.

Nanofibrous scaffolds made from gelatin have also been developed and may have a promising future in cartilage tissue regeneration. Agheb et al. [10] developed electrospun gelatin fiber meshes that were crosslinked before or after synthesis with glutaraldehyde or EDC/NHS chemistry. To increase the functionality of these scaffolds, the authors also embedded tyrosine and triazole rings since an increase in aromatic ring content has also been shown to enhance the tensile strength and bioactivity of materials. Fibers crosslinked after electrospinning exhibited larger diameters, reduced porosity, greater rigidity, and smaller pore sizes. As a result, those crosslinked before electrospinning sustained better chondrocyte proliferation and viability in later tests. The incorporation of additional aromatic rings was also shown to increase chondrocyte viability and allowed them to express their natural phenotype and morphology, leading the authors to believe that the modified gelatin scaffolds could be used to promote cartilage regeneration in vivo.

These above studies illustrate that protein-based nanofibers can be used to promote regeneration of a variety of tissue in nanoscale. In all cases, nanofibers were easily fabricated and modified by chemical functionalization, crosslinking, or polymer blending showcasing the versatility of such materials.

### 4.2. Drug Delivery

Nanofiber materials are often used in drug delivery since their high surface-area-to-volume ratio and porosity allow for high efficiency drug storage and release. Protein-based nanofibers have particular appeal over their synthetic counterparts since the materials are biodegradable and biocompatible. They have also been shown to be highly modifiable, allowing researchers to tune the release of pharmaceuticals.

**Figure 8:**
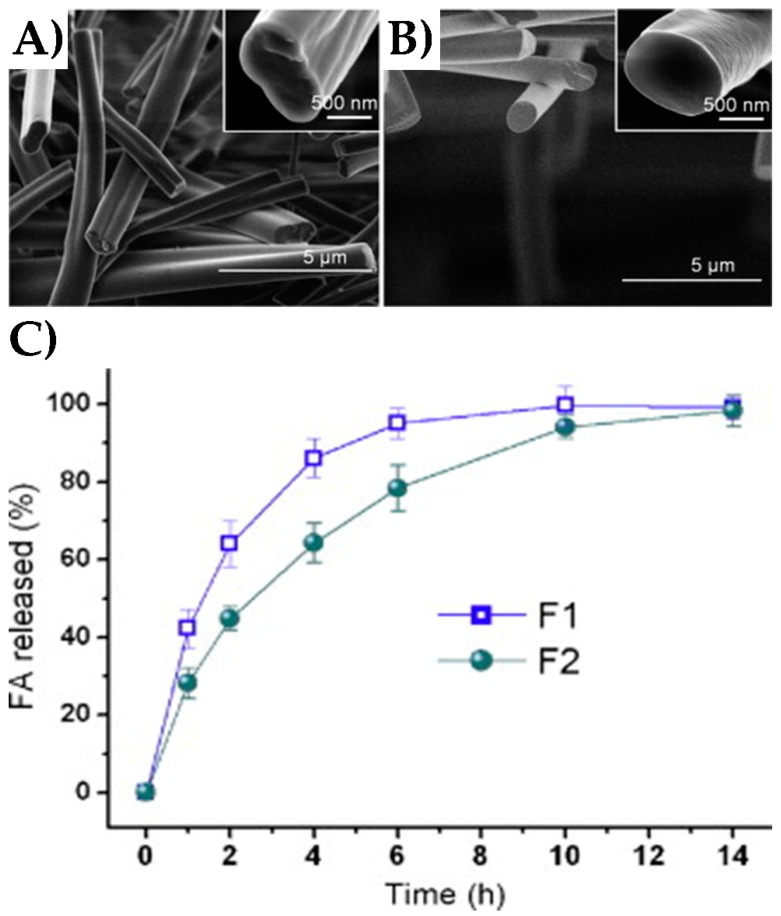
Corn zein nanofibers containing ferulic acid (FA) can be fabricated through single-fluid electrospinning (**A**) and modified coaxial electrospinning (**B**). Release of FA from both fiber types (F1 and F2) was then monitored over time (**C**) (reproduced with permission from [173], Copyright Elsevier, 2013).

Due to the biodegradability, flexibility, biocompatibility, anti-microbial properties, and the anti-oxidant behavior of corn zein protein, it is commonly used to create nanofibers that are used in drug delivery [19,172]. Figure 8 shows the results of a drug delivery study done with co-axial electrospun corn zein nanofibers [173]. In coaxial electrospinning, two liquids are spun simultaneously from one concentric spinneret to create fibers with a core-sheath structure. However, to generate higher quality fibers with increased smoothness and uniformity, an unspinnable solvent containing no polymer was used to create the sheath, while corn zein was used to create the core. This unspinnable solvent, in this case dimethylformamide (DMF), is unable to form nanofibers, but replaces the standard polymer-air interface usually seen during electrospinning. In this way, an air-solvent-polymer interface is established, thus mitigating environmental effects on fiber formation. Ferulic acid (FA) was incorporated into the corn zein-ethanol solution prior to spinning and served as the model drug. Corn zein nanofibers created through standard electrospinning (without the unspinnable solvent sheath) were designated F1, while those made with coaxial spinning were designated F2 (Figure 8). As seen in Figure 8A, FA is released from both types of electrospun corn zein fibers through a standard Fickian diffusion mechanism. However, F2 fibers exhibited a longer, more controlled release than F1. This was attributed to the fact that F1 fibers had a flatter, more ribbon-like structure than F2 due to the coaxial spinning mechanism. F1 fibers also had a more wrinkled appearance than F2. Both of these characteristics contribute to a higher surface area on the F1 fibers, leading to faster, burst release of FA. This study illustrates the use of corn zein nanofibers as drug delivery vehicles and also the ability to optimize fiber fabrication methods to achieve the desired release profiles of drugs.

Soy protein has also been used to create fiber meshes for applications in drug delivery. Xu et al. [174] developed soy protein microfibers by dissolving the protein in an aqueous urea solution and extruding it into a sodium sulfate solution. This produced fibers with an average diameter of 45 µm. Model drugs, including diclofenac, 5 fluorouracil (5-Fu), and metformin, were incorporated into fibers prior to spinning or loaded into fibers through sorption by exposing the dry fibers to solutions containing drugs at various conditions. Overall, the researchers found that the soy protein fibers had a high affinity to the model drugs, allowing for efficient sorption loading. This loading could also be modified by modulating the temperature during fiber exposure. Burst release could also be limited by lowering the concentration of the loaded drug.

Like those used in tissue engineering, natural and synthetic polymer composite fibers can also be used for drug release. For example, Lee et al. [175] developed multi-layered PLGA/collagen nanofibers membranes through electrospinning, that were used to deliver lidocaine and epinephrine over four weeks. These membranes were then used to deliver the drugs to rabbits with palatal oral wounds and test groups showed faster hemostasis, as well as recovery of food and water intake, compared to control groups who received an empty PCL membrane. In place of a model drug, proteins have also been added to synthetic polymer-based nanofibers and released over time. Zeng et al. [176] prepared these protein-loaded fibers by electrospinning a solution of poly(vinyl alcohol) and bovine serum albumin protein (BSA). To control the release of BSA, the fibers were also coated with poly(p-xylylene) (PPX). This coating was found to slow the release of BSA over 20 days, and successful release of the model enzyme luciferase was also observed. This demonstrates that proteins can be easily incorporated into fibers for structural purposes, as well as therapeutic ones.

### 4.3. Wound Healing

Due to their porosity, gas permeability, and high surface-area-to-volume ratio, fibrous materials may offer advanced treatment options for burn victims and patients with skin ulcers compared to conventional treatment options like hyperbaric oxygen therapy. A successful wound dressing is one that is able to facilitate epithelial cell migration and regeneration, and this is often best achieved by creating a warm, moist environment. During healing, it is also important to prevent the influx of bacteria that can lead to an infection that delays wound healing. To meet these requirements, protein-based fiber meshes are commonly used for the creation of conventional bandages, antimicrobial-infused dressings, and advanced tissue engineered skin grafts [177,178].

**Figure 9:**
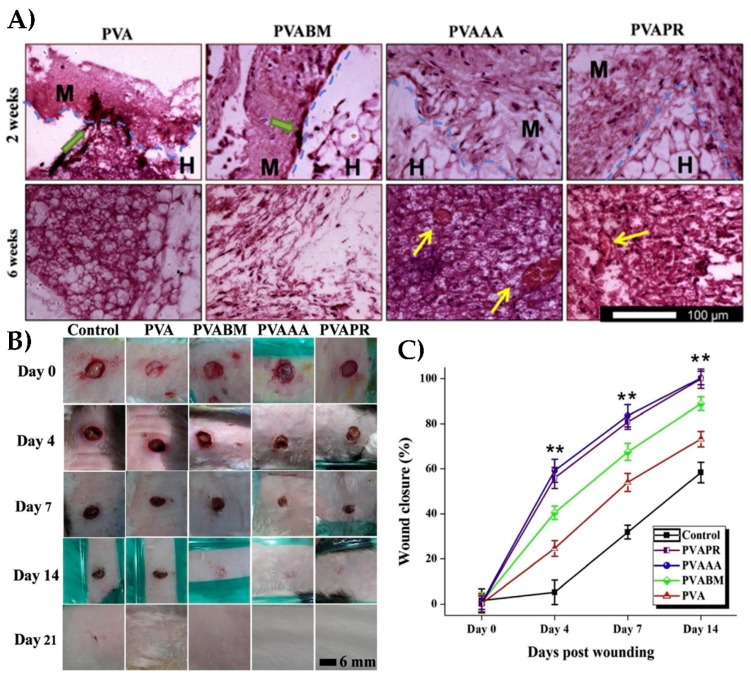
(**A**) Hematoxylin and eosin staining for histological analysis of subcutaneously implanted mats made of varying PVA-silk nanofiber composite fibers. Mats containing non-mulberry silk showed greater cell infiltration and proliferation compared to those made of mulberry silk or those containing only PVA. Green arrows indicate infiltrating cells at the interface of the implant, and yellow arrows indicate the development of new blood vessels. (**B**) Images of wounds on rabbits treated with PVAA-silk mats. The percentage of wound closure is quantified in (**C**) (reproduced with permission from [25], Copyright Elsevier, 2017).

In an effort to promote wound healing and minimize infection and inflammation Chouhan et al. [25] recapitulated the physical and biological ECM of dermal tissue by creating electrospun silk fibroin meshes with varying concentrations of poly(vinyl alcohol) (PVA) for added mechanical stability. Silk fibroin from the *Bombyx mori* (PVAABM) silkworm was used, as well as non-mulberry silk from *Antheraea assama* (PVAAA) and *Philosamia ricini* (PVAPR). These meshes were then functionalized with epidermal growth factor (EGF) and ciprofloxacin HCl antibiotic. All fibers allowed burst release of EGF, but those containing silk fibroin achieved a greater release over 24 h, compared to those made solely from PVA. Since non-mulberry silk fibroin contains a naturally occurring RGD motif, these fiber meshes were also able to support greater cell adhesion and proliferation, as seen in Figure 9A. Silk fibroin also allowed for greater water retention, allowing for the creation of the moist environment, crucial in wound regeneration. Due to these characteristics, nanofiber meshes containing silk fibroin resulted in faster wound closure than meshes containing PVA alone, as seen in Figure 9B,C. Meshes containing non-mulberry silk fibroin out-performed those containing *B. mori* silk fibroin with 100% closure being achieved after 14 days, compared to only 80% closure in the latter samples.

In addition to growth factors and antibiotics, nanoparticles can also be embedded into protein-based fiber meshes. This is advantageous, since some metal nanoparticles, such as those made from silver (AgNPs), can have antimicrobial properties. Wang et al. [179] incorporated AgNPs into keratin-based nanofibers that were blended with polyurethane and fabricated through the electrospinning process. When used to heal circular wounds in rats, these nanofiber meshes resulted in 30% wound closure after nine days, compared to only 60% wound closure in a control group that received conventional sponge dressing. A reduction of TNF-α secretion and inflammatory cell infiltration was also seen. The incorporation of keratin into poly(hydroxybutylate-cohydroxyvalerate) (PHBV) nanofiber mats was also shown to accelerate the proliferation of human fibroblast cells, as demonstrated by Yuan et al. [180].

## 5. Conclusions

The use of fibrous materials in biomedical research has been growing in popularity due to their high surface-area-to-volume ratio, tunability, porosity, and mechanical strength. Fibers made from natural polymers, such as proteins, hold additional promise due to their biodegradability and biocompatibility. Natural enzymes within the body are able to degrade these proteins to produce amino acids that pose no risk of toxicity and can be reabsorbed. Proteins, such as silk, collagen, and keratin, also contain innate cell adhesion motifs. Therefore, when these constructs are used in tissue engineering or wound healing applications, they can support increased cell migration and proliferation. The mechanical and physical properties of many protein-based fiber materials can also be modified by crosslinking or blending an additional polymer, creating a tunable platform. These characteristics are also highly dependable on the type of the fabrication method used to create fibers. While electrospinning is currently the most popular technique, other methods, such as wet/dry jet spinning, dry spinning, centrifugal spinning, solution blowing, self-assembly, phase separation, and drawing, have been successful. As biomedical research and technology progresses, protein-based fibers may lead the way in the development of new biomaterials that promote tissue regeneration, wound healing, and controllable drug delivery.

## Figures and Tables

**Table 1:** Parameters of fabrication methods affecting fiber properties [118,119,120,121,122,123,124,125,126,127,128,129,130].

Fiber Fabrication Method	Parameters to Control Fiber Formation		
	Solution	Process	Environment
Electrospinning	- Polymer concentration - Viscosity - Conductivity - Solvent evaporation rate - Molecular weight	- Flow rate - Applied voltage - Tip to collector distance - Collector types	- Temperature - Humidity
Wet-/Dry-Jet Spinning	- Polymer concentration - Viscosity - Molecular weight	- Coagulation medium - Coagulation bath concentration - Post-drawing	- Temperature - Humidity
Dry Spinning	- Polymer concentration - Molecular weight	- Post-drawing - Take up speed	- Temperature - Humidity
Centrifugal Spinning	- Viscosity - Surface tension - Molecular structure - Molecular weight - Polymer concentration - Solvent structure or evaporation rate - Additive	- Rotational speed - Head diameter - Nozzle diameter - Distance from nozzle to collector	- Temperature - Humidity
Solution Blowing	- Polymer type - Concentration - Solvent evaporation rate - Molecular weight	- Injection rate - Gas flow pressure - Distance from nozzle to collector	- Temperature - Humidity

**Table 2:** Overview of applications of protein-based nanofibers in medicine.

Protein	Fabrication Technique	Material	Application
Keratin	Electrospinning	AGNP-embedded keratin-Polyurethane nanofibers PHBV-keratin nanofiber mats	Dermal wound healing [179] Fibroblast cell proliferation [180]
Collagen	Electrospinning	Crosslinked collagen nanofiber meshes PCL-collagen nanofiber meshes PCL-collagen nanofiber meshes embedded in hyaluronic acid hydrogel PLGA-collagen nanofibers	Bone tissue regeneration [167] Aortoiliac bypass [168] Vascularized bone regeneration [169] Delivery of lidocaine and epinephrine [175]
Gelatin	Electrospinning	Crosslinked gelatin nanofibers with embedded tyrosine and triazole rings	Cartilage regeneration [10]
Silk Fibroin	Electrospinning	Silk fibroin nanofiber meshes PEUU-silk fibroin nanofiber mashes EGF-functionalized PVA-silk fibroin nanofibers	Bone tissue regeneration [171] Heart valve regeneration [87] Dermal wound healing [25]
Zein	Co-axial Electrospinning	Corn zein nanofibers	Delivery of ferulic acid [173]
Soy	Wet-spinning	Soy protein microfibers	Delivery of diclofenac, 5 fluorouracil and metformin [174]
Elastin	Electrospinning	Crosslinked PLGA-elastin nanofiber meshes	Regeneration of epithelial constructs [18]
